# Establishment and characterization of a cell line (NOMH-1) originating from a human endometrioid adenocarcinoma of the ovary

**DOI:** 10.1186/1757-2215-6-8

**Published:** 2013-02-04

**Authors:** Takashi Yamada, Takayoshi Kanda, Hiroshi Mori, Kaname Shimokawa, Mitsuo Kagawa, Yuro Shibayama

**Affiliations:** 1 Department of Pathology, Osaka Medical College, 2-7 Daigaku-machi, Takatsuki, Osaka, 569-8686, Japan; 2 Department of Obstetrics and Gynecology, Osaka Minami Medical Center, 2–1 Kidohigashi-machi, Kawachinagano, Osaka, 586-8521, Japan; 3 Department of Clinical Pathology, Minoh City Hospital, 5-7-1 Kayano, Minoh, Osaka, 562-8562, Japan

**Keywords:** Endometrioid adenocarcinoma, Ovary, Cell line, Chemosensitivity, MTT assay, Tumor marker

## Abstract

**Background:**

Cell lines are very useful for clinical and basic research. Thus far, only 11 reports have documented the characteristics of ovarian endometrioid adenocarcinoma cell lines in the literature. Due to the scarcity of information, the establishment of an ovarian malignant tumor cell line with distinctive characteristics is particularly important to study this disease. Thus, this study was undertaken to establish and characterize a new human endometrioid adenocarcinoma cell line of the ovary.

**Methods:**

The cell line NOMH-1 was established from an ovarian tumor of a 44-year-old woman. Features of the cell line studied included morphology, chromosome analysis, heterotransplantation, tumor markers, and chemosensitivity.

**Results:**

This cell line has been growing well for 232 months and subcultured more than 50 times. Monolayer cultured cells were polygonal in shape, showing a pavement-like arrangement and a tendency to stack without contact inhibition. They exhibited a human karyotype with a modal chromosomal number in the hypertriploid range. The cells could be transplanted into the subcutis of nude mice and produced tumors resembling the original tumor. NOMH-1 cells expressed both CEA and CA19-9 which were identified immunohistochemically in the original tumor and the heterotransplanted tumor. The cells were sensitive to paclitaxel, an agent commonly used in the treatment of gynecological cancers.

**Conclusions:**

NOMH-1 is the first ovarian endometrioid adenocarcinoma cell line in which CEA and CA19-9 expression have been defined. This newly established cell line should be useful for investigating the characteristics of ovarian endometrioid adenocarcinoma.

## Background

Endometrioid adenocarcinoma of the ovary is a variant of epithelial ovarian cancer and has frequent concurrence with endometriotic lesions. Recent studies suggest that most endometrioid cancers arise from endometriosis [1,2], and they are sometimes associated with carcinoma of the endometrium [3]. Therefore, it was considered important to develop an ovarian endometrioid adenocarcinoma cell line for clinical and basic research of this disease. We describe here the establishment and characterization of a new human cell line (NOMH-1) of ovarian endometrioid adenocarcinoma that express both carcinoembryonic antigen (CEA) and CA 19–9.

## Methods

### Patient and clinical background

**Figure 1:**
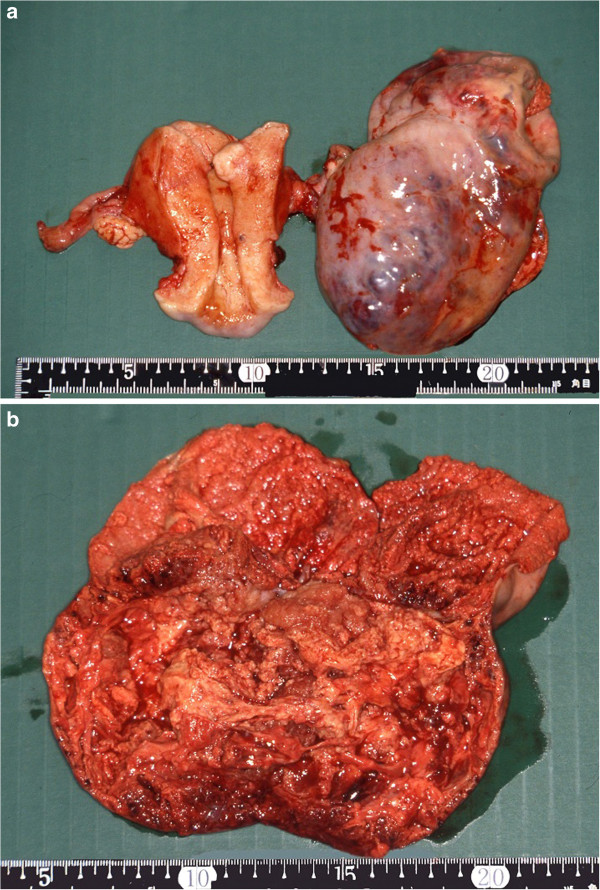
**Macroscopic appearance of the original tumor (a, b). **The left ovary was a solid tumor.

On June 30, 1993, we performed an abdominal simple total hysterectomy, bilateral salpingo-oophorectomy, and omentectomy on a 44-year-old woman with ovarian cancer FIGO stage IIc (Figure 1). The patient gave informed consent for performance of this study. Histology was performed on the resected tissue, and the tumor was diagnosed as an endometrioid adenocarcinoma of the ovary by intraoperative diagnosis of frozen section. Assessment of preoperative serum tumor marker levels showed that CA 125 was 639 U/ml (normal level <35 U/ml), CA 19–9 was 108 U/ml (<37 U/ml), and CEA was 2.4 ng/ml (<2.5 ng/ml). The patient was treated twice with 100 mg cisplatin (CDDP) and 400 mg etoposide (VP-16) intraperitoneally, and 5 times with 450 mg carboplatin (CBDCA), 60 mg pirarubicin hydrochloride (THP), and 400 mg cyclophosphamide (CPA) intravenously. However, a follow up examination on March 25, 1994 showed that her serum tumor markers were increased: CA 125 was 158 U/ml, CA 19–9 was 438 U/ml, and CEA was 6.3 ng/ml. Her clinical condition deteriorated progressively because of a recurrent abdominal tumor, and she died on October 11, 1994. 

### Culture techniques and culture media

Tissue from the resected ovarian tumor was finely minced with a pair of sharp blades in a dish including serum-free Ham’s F-12 medium (Flow Laboratories Inc., McLean, VA), stirred slowly with a magnetic stirrer in a 0.25% trypsin solution (Flow Laboratories Inc.), centrifuged at 70 g for 5 min, and placed in culture medium at 37°C in humidified 5% CO_2_ and 95% air. Cells were cultured in Ham’s F-12 medium plus 20% precolostrum newborn calf serum (Mitsubishi Chemical Industries Ltd., Tokyo, Japan) with kanamycin. Then, subcul-tures were passaged with 0.1% trypsin and 0.02% ethylenediamine-tetraacetic acid (EDTA) solution every 4 weeks. Six months after the primary culture, the concentration of precolostrum newborn calf serum in the culture medium was reduced from 20% to 10%.

### Morphology of the resected original tumor and cultured cells

Living cells grown in culture flasks were examined using a phase contrast microscope. For histology, the original tumor was fixed in 10% formalin, embedded in paraffin, and 4 μm-sections were stained with hematoxylin-eosin (HE). Monolayer cells cultured on slides were fixed in 90% ethanol and stained by Papanicolaou’s procedure [4]. For electron microscopy, the original tumor was fixed by immersion in a mixture of 1.25% glutaraldehyde and 1% paraformaldehyde buffered with phosphate saline, pH 7.4, at 4°C for 3 hr. After washing with phosphate buffered saline (PBS), the tumor was postfixed with 1% osmium tetroxide at 4°C for 1 hr, then washed in PBS, dehydrated in graded concentrations of ethanol, and embedded in Epon 812. Sections 0.5 μm thick were cut with a 6000 ultramicrotome (Sorvall, Du Pont, CT) and stained with toluidine blue. Ultrathin sections exhibiting light gold interference color were cut from the corresponding areas in the toluidine blue stained sections, double stained with uranyl acetate and lead citrate, and observed under a JEM-100SX electron microscope (JEOL, Tokyo, Japan) at 80 kV [5].

### Growth characteristics

Cell characteristics were examined in passages 5–10. Suspensions of 1 × 10^5^ cells were plated in 35 mm plastic dishes and incubated for 44 days. Then, cells from two dishes were counted every other day by an automatic cell counter (Coulter Counter^R^, Coulter Electronics, Luton, England). The population doubling time and saturation density were calculated from the growth curve. For studies of plating efficiency, 1×10^2^ and 2×10^2^ single-suspension cells were placed into five 60 mm plastic dishes each and cultured for 21 days. Plating efficiency was determined by the ratio of the number of colonies (more than 10 cells) to the total number of inoculated cells. For the mitotic index, monolayer cells were cultured for 5 days and treated with 1×10^-7^ M colcemid (Demecolcine Solution, Wako Pure Chemical Industries, Osaka, Japan) for 4 hr, placed in a 0.2% KCl solution for 15 min, and then fixed step-by-step in a methanol: acetic acid solution (3:1). After air-drying, the cells were stained with Giemsa and the number of mitotic cells in 1,000 cells were counted.

### Chromosome analysis

Histograms of chromosome number distribution were determined using 39 metaphase plates. Their karyotypes were analyzed in 10 cells in accordance with the International System for Human Cytogenetic Nomenclature (ISCN 1995).

### Heterotransplantation

Ten million cells (passage 10) were inoculated subcutaneously into the dorsal region of 6-week-old nude mice (BALB/cAJcl-nu; Clea Japan, Inc., Tokyo, Japan). When the tumors had grown to 5–10 mm in diameter after 4 weeks, they were removed and processed for morphological examinations. For histology, the excised tumors were fixed in 10% formalin, embedded in paraffin, and stained with HE. For electron microscopy, the tumors were cut into pieces with a pair of sharp blades fixed, stained, and observed as described above.

### Tumor markers

Culture medium in which 2×10^6^ cells / 5 ml were cultured for 7 days was examined for α fetoprotein (AFP), CA 125, CA 19–9, CA 72–4, CEA, human chorionic gonadotropin (HCG), squamous cell carcinoma (SCC) antigen, and tissue polypeptide antigen (TPA) by radioimmunoassay or chemiluminescent immunoassay.

### Immunohistochemical stainings

Deparaffinized sections of the original and heterotransplanted tumors on glass slides were immunohistochemically stained using the universal Immuno-enzyme Polymer (UIP) method (Envision kit; DAKO, Glostrup, Denmark). Slides were immersed in 0.03% hydrogen peroxidase and absolute methanol to block endogenous peroxidase, and then washed with phosphate buffered saline and heated in an autoclave for 20 min. After cooling, the slides were incubated with a primary antibody at room temperature for 40 min and then reacted with Envision (DAKO) at room temperature for 30 min, followed by incubation with diaminobenzidine (DAB) for 5–10 min. Antibodies against CA 125 (DAKO), CA 19–9 (DAKO), CEA (DAKO), and P53 (DAKO) were used to detect tumor markers, and against human estrogen receptor α (Nichirei Bioscience Inc., Tokyo, Japan) and human progesterone receptor (Nichirei) to detect hormone receptors.

### Chemosensitivity assays

The effects of actinomycin D (ACD, MSD KK, Tokyo, Japan), doxorubicin (ADM, Kyowa Hakko Kirin Co., Ltd., Tokyo, Japan), 5-fluorouracil (5-FU, Kyowa Hakko Kirin Co.), mitomycin C (MMC, Kyowa Hakko Kirin Co.), bleomycin (BLM, Nippon Kayaku Co., Ltd., Tokyo, Japan), vinblastine (VLB, Nippon Kayaku Co.), vincristine (VCR, Nippon Kayaku Co.), carboplatin (CBDCA, Bristol-Myers K.K., Tokyo, Japan), cisplatin (CDDP, Bristol-Myers), etoposide (VP-16, Bristol-Myers), paclitaxel (PTX, Bristol-Myers) [6], 4-hydroperoxy-cyclophosphamide (CPA, Shionogi & Co., Ltd., Osaka, Japan), irinotecan SN-38 (CPT-11, Yakult Honsha Co., Ltd., Tokyo, Japan) [7], and methotrexate (MTX, Pfizer Japan Inc., Tokyo, Japan), which are often used to treat gynecological cancers [8], on the cultured cells were investigated by 3-(4,5-dimethyl-2-thiazolyl)-2,5-diphenyl-2H tetrazolium bromide (MTT) assay. The drugs were dissolved in culture medium and used immediately. For MTT assay, 5 x 10^3^ cells in 100 μl medium were seeded in tetraplicate into each well of 96-well microwell plates. For continuous drug exposure experiments, various diluted drugs in 50 μl were added after 48 hr of incubation. The wells were incubated for 72 hr after the addition of drugs. MTT (50 μl of 2 mg/ml; Wako Pure Chemical Industries, Ltd, Osaka, Japan) was added to each well, and the plates were incubated for 4 hr. The medium was then removed, 150 μl of dimethyl sulfoxide (DMSO, Sigma, St. Louis, MO) was added to each well, and the plates were agitated for 5 min. The optical density was then read at 570 nm on a microplate reader (Bio-Rad Laboratories, Hercules, CA) and the effective concentration of the median lethal dose was calculated from the dose response curve (EC50: dose of drug required to reduce final cell number or optical density in MTT assay to 50% of control) [9].

## Results

### Histopathology of the original tumor specimen

**Figure 2:**
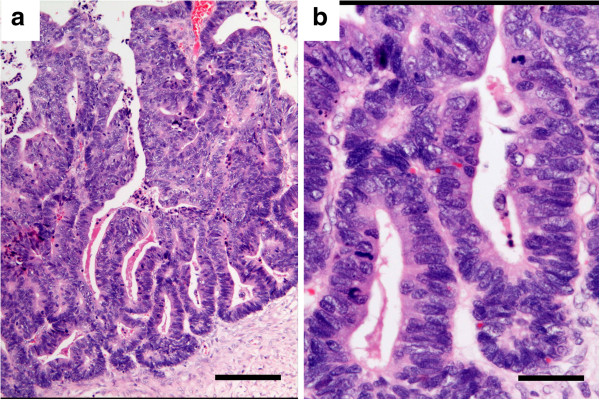
**Histology of the original tumor. **It shows an endometrioid adenocarcinoma stained with hematoxylin and eosin (HE). (a,bar = 200 μm; b,bar = 50 μm).

Light microscopy revealed the original tumor to be an endometrioid adenocarcinoma, which showed tubular glands lined by stratified non-mucin containing epithelium (Figure 2). 

### Establishment of the cell line

Tissue fragments from the original tumor were cultured and after a 2-month stationary period, definite out-growths developed. Initially, contamination by spindle-shaped fibroblasts was observed, but they disappeared from the cultures upon passaging the cells, which were named NOMH-1. The NOMH-1 cells grew well, without interruption, for more than 232 months, and more than 50 serial passages were successively carried out. They continue to exhibit stable growth.

### Morphology of the cultured cells

**Figure 3:**
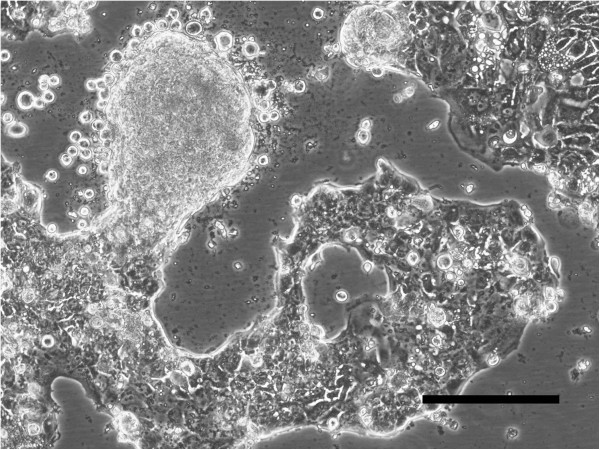
**Phase contrast microscopy. **The cells show a pavement-like arrangement and colonies with multilayering. (bar = 200 μm).

**Figure 4:**
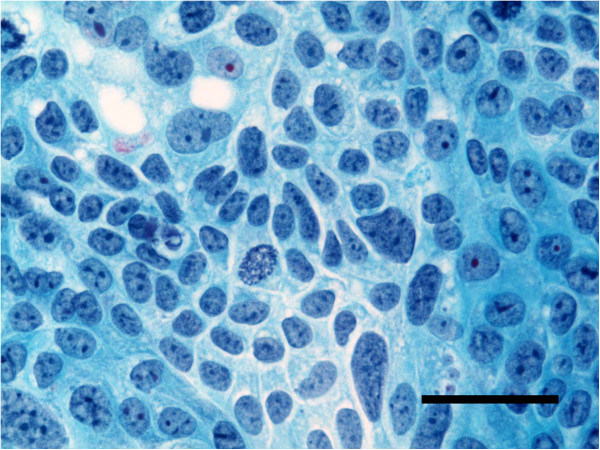
**Cytopathology of cultured NOMH-1 cells. **Multinucleated giant cells with spindle-shaped or multipolar cytoplasm are observed by Papanicolaou stain. (bar = 50 μm).

**Figure 5:**
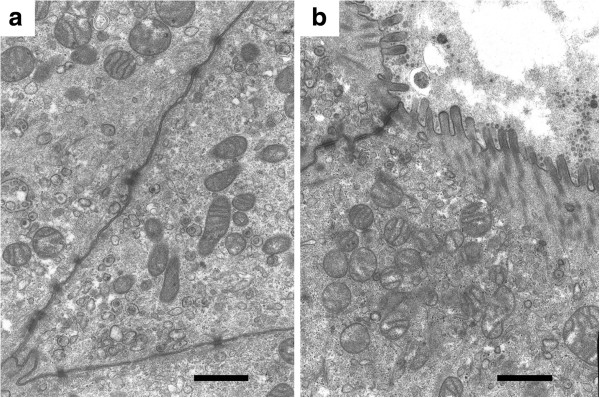
**Electron micrograph of the original tumor. **The cells are attached by desmosome-like junctions (**a**) and have abundant mitochondria in the cytoplasm (**a**)(**b**) and numerous microvilli on the surface (**b**). (a,b, bar = 1 μm).

The cultured cells grew in monolayer and appeared to be epithelial, showing a pavement-like arrangement. Multilayering of cells was easily observed even before they reached confluency (Figure 3). The cells were polygonal and showed neoplastic features such as bizarre aggregation of chromatin granules, thickened nuclear membrane, and multiple large nucleoli. Multinucleated giant cells were also seen (Figure 4). Electron microscopy revealed that adjacent cells showed desmosome-like junctions (Figure 5a). The cells had indented nuclei (figure not shown), abundant mitochondria in the cytoplasm, and numerous microvilli on the cell surface (Figure 5b). These features suggested that the cells were epithelial in origin. 

### Growth characteristics

**Figure 6:**
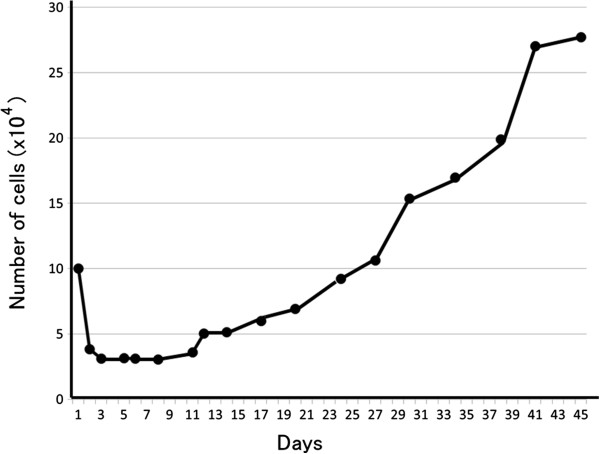
**Growth curve of NOMH-1. **The cells grew logarithmically.

The growth curve was examined in passage 5 of the NOMH-1 cell line. Three days after culturing, the cells grew logarithmically (Figure 6). The population doubling time, saturation density, plating efficiency, and mitotic index were 273 hr, 3.7 × 10^4^ cells/cm^2^, 3.6%, and 4.9%, respectively. 

### Chromosome analysis

**Figure 7:**
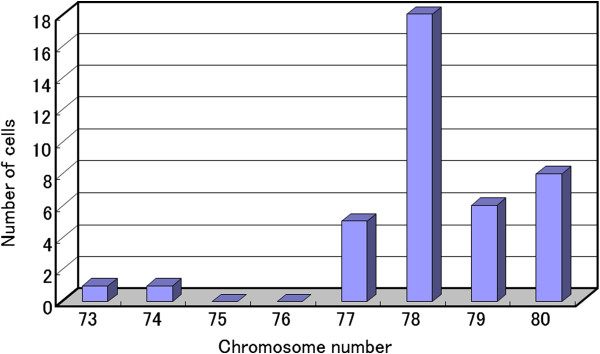
**Distribution of chromosomal numbers in NOMH-1 (5th generation). **The modal number is in the hypertriploid range.

**Figure 8:**
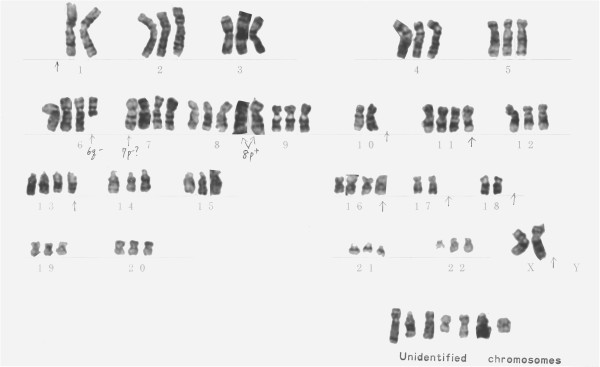
**Karyotype of NOMH-1 (5th generation). **Chromosomal analysis showed various abnormalities.

This cell line had a modal chromosome number that was in the hypertriploid range (73–80) (Figure 7). Chromosomal analysis showed that all of the cells had 6q- and most of the cells had 8p+ (Figure 8). 

### Heterotransplantation

**Figure 9:**
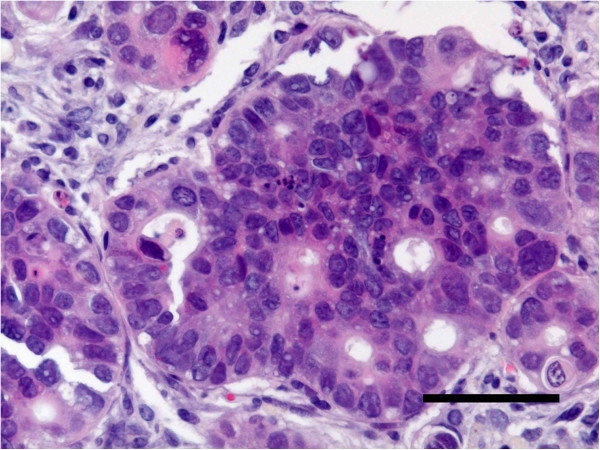
**Micrograph of a tumor transplanted into a nude mouse. **It shows endometrioid adenocarcinoma closely resembling the original tumor. (HE, bar = 50 μm).

Histopathologically, the transplanted tumors were endometrioid adenocarcinomas that showed tubular glands lined by stratified non-mucin containing epithelium, which closely resembled the original tumor (Figure 9). Electron microscopy showed desmosome-like junctions and numerous microvilli as observed in the original tumor. 

### Tumor markers

**Figure 10:**
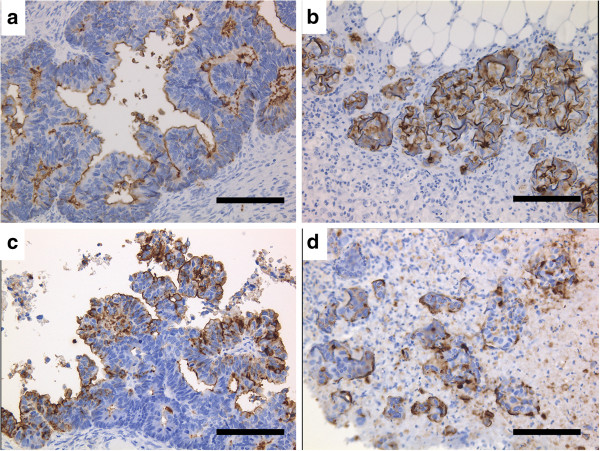
**Immunohistochemical stainings of the original tumor (a, c) and heterotransplanted tumor (b, d) with CEA (a, b) and CA19-9 (c, d). **(a, b, c, d; bar = 100 μm).

This cell line was positive for the following tumor markers: CEA, 1,000 ng/ml; CA 19–9, 305 U/ml; CA 72–4, 5.0 U/ml; and TPA, >1,500 U/L. On the other hand, they were negative for: AFP, <1 ng/ml; CA 125, 20 U/ml; HCG, <1 mIU/ml; and SCC antigen, <0.5 ng/ml. Expression of CEA and CA 19–9 was demonstrated immunohistochemically in the cancer cells of the original and heterotransplanted tumors (Figure 10). In contrast, CA125 and P53 were negative immunohistochemically. 

### Hormone receptors

Neither estrogen receptor (ER) nor progesterone receptor (PgR) were detected immunohistochemically in the original and heterotransplanted tumors.

### Chemosensitivity

**Figure 11:**
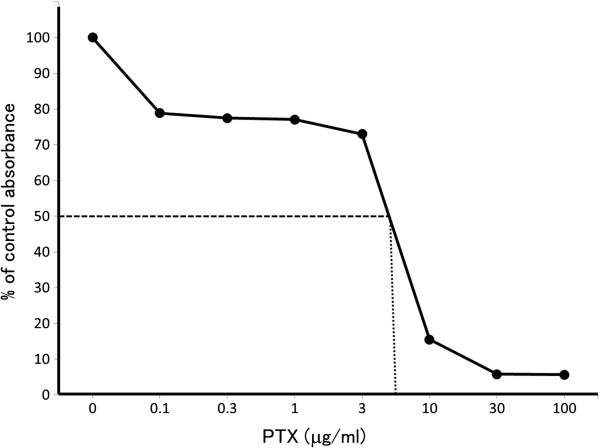
**Dose response curve for paclitaxel. **The EC50 value of paclitaxel is 5.1.

The EC50 values of anti-cancer drugs for NOMH-1 cells are listed in Table 1 (Figure 11). 

**Table 1:** Chemosensitivity using a 3-(4,5-dimethylthiazol-2-yl)-2,5-diphenyl tetrazolium bromide assay

**Drug**	**EC50**	**PPC**
	**(μg/mL)**	**(μg/mL)**
ACD	>0.1	0.08
ADM	16.5	0.4
BLM	>1000	3.3
CBDCA	>300	37.1
CDDP	23.2	8.5
CPA	>10	4.3
CPT-11	1.79	0.05
5-FU	497	15.3
MMC	6.5	2.4
MTX	>300	3
PTX	5.1	11.8
VCR	15	0.1
VLB	16	0.15
VP-16	197	13

EC50, effective concentration for 50% kill; PPC, peak plasma concentration taken intravenously; ACD, actinomycin D; ADM, doxorubicin; BLM, bleomycin; CBDCA, carboplatin; CDDP, cisplatin; CPA, 4-hydroperoxy-cyclophosphamide; CPT-11, irinotecan hydrochloride; 5-FU, 5-fluorouracil; MMC, mitomycin C; MTX, methotrexate; PTX, paclitaxel; VCR, vincristine; VLB, vinblastine; VP-16, etoposide;.

## Discussion

In this study, we established a novel cell line, NOMH-1, of human endometrioid adenocarcinoma of the ovary by culturing tissue fragments from a resected tumor, and to obtain convincing evidence that this cell line truly reflects the original tumor and disease, we investigated its biological characteristics. We found that these cells showed the following features: 1) viable in culture for over 232 months; 2) neoplastic, pleomorphic, and stack easily without contact inhibition; 3) chromosomes were of a human karyotype with aneuploid distribution; and 4) transplantable into nude mice and formed tumors which histologically resembled the original tumor.

Despite the advances in culturing technology, it is still difficult to establish a cell line, chiefly because of contamination by fibroblasts, which usually grow faster than epithelial cells and promote their detachment from the plate. To overcome this, we took advantage of the fact that fibroblasts detach faster than epithelial cells by trypsin, and we removed every fibroblast and most epithelial cells, leaving only a few colonies of epithelial cells to grow. The cells gradually grew well to form obvious colonies, and the first subculture was made 4 months after the primary culture.

Some ovarian endometrioid adenocarcinoma cell lines have been used for basic research, but only eleven of them (including #2774 [10], endometrioid carcinoma cell line [11], OVK18 [12], HMOA [13], HNOA [13], HOC-I [14], COV362 [15], SIB-1 [16], SNU-251 [17], SNU-563 [17], and NOE [18] ) have had their characteristics described in detail in the literature (Table 2). Each cell line originated from an original tumor, recurrent tumor, ascites, pleural effusion, or tumor fluid. It seemed that cancer cells in the fluid were more easily cultured. The population doubling time varied greatly (28–75 hr), the modal chromosome numbers were in the diploid range or more, and all the cells showed karyological abnormalities. Our NOMH-1 cells grew well and detached easily after multilayering, therefore, its population doubling time seemed longer than other cell lines. 

**Table 2:** Cell lines from ovarian endometrioid adenocarcinoma

**Cell line**	**Age**	**Materials**	**Chromosome Number**	**DT**	**Transplantability**	**Immunotaining**	**Characteristics**
#2774 (1978)	-	ascites	66-68	-	Yes	-	-
- (1983)	47	ascites	103 (51–402)	74.4	Yes	-	-
OVK18 (1983)	49	ascites	47 (mode)	48	Yes	-	ER(−), PgR(−)
HMOA (1986)	57	ovarian tumor	46-47 (mode)	72, 58	Yes	-	production of CA125
HNOA (1986)	71	transplanted tumor	46-47 (mode)	28, 24	Yes	-	CA125(−)
HOC-I (1989)	40	recurrent tumor	46 (44–49)	75	Yes	ER(−)	ER(−), production of CA125, TPA
COV362 (1993)	-	-	69 (67–73)	-	-	-	-
SIB-1 (1994)	39	pleural effusion	46 (43–54)	-	-	HMFG-2, CA125	serum free medium
SNU-251 (1997)	47	ascites	-	46	-	-	CA125(−), CEA(−), p53, BRCA1, hMLH1 mutation(+)
SNU-563 (1997)	54	ovarian tumor	-	67	-	-	p53 mutation(+)
NOE (2007)	46	tumor fluid	65-74	-	Yes	-	ER(+)
NOMH-1	44	ovarian tumor	73-80	273	Yes	CA19-9, CEA, ER(−), PgR(−)	production of CA19-9, CEA, TPA

DT; doubling time (hour).

While two previously reported cell lines were shown to produce CA125, only one was demonstrated immunohistologically. Tumor markers are useful not only in diagnosing ovarian cancer but also in detecting tumor recurrence or assessing therapy. In this patient, CEA and CA19-9 were especially useful tumor markers because her serum levels were postoperatively high, which were reproduced in the cultured cells. NOMH-1 is the first cell line of ovarian endometrioid adenocarcinoma which produces both CEA and CA 19–9. Therefore, it represents a good model for the study of ovarian cancers that express tumor markers.

The ovary is considered a target organ of steroid hormones, and while NOE expresses estrogen receptor, OVK18, HOC-I, and NOMH-1 produce neither estrogen receptor nor progesterone receptor. As the mechanism of action and localization of steroid hormone receptors are still largely unknown, NOMH-1 can be used as a non-hormone receptor cell line for studies on receptors.

Chemotherapy as well as surgery are very important modalities for the treatment of ovarian cancer. The CAP (CPA, ADM, CDDP) regimen had been commonly used for treatment of ovarian epithelial carcinoma, however, PTX and CBDCA have been recently used although they are not always effective. Thus, CPT-11 has been used for platinum- and taxanes-resistant epithelial ovarian cancer [19]. In order to determine the effects of chemotherapeutic reagents on NOMH-1 cells, chemosensitivity to a panel of drugs was measured using the MTT assay, which is still considered a rapid and accurate method of screening for drug responsiveness of cultured cells. *In vitro* sensitivity was defined as more than 50% growth inhibition at peak plasma concentrations. According to this criterion, we demonstrated that NOMH-1 cells were sensitive to PTX only. Unfortunately, PTX could not be used to treat the patient at that time, which reflected her severe clinical course.

Since it is impossible to establish a cell line from the malignant tumor of each patient, the cell line that we established and characterized would be very useful in basic research on ovarian cancer, especially endometrioid adenocarcinoma, the pathogenesis of which is not yet completely known.

## Abbreviations

CEA: Carcinoembryonic antigen; CDDP: Cisplatin; VP-16: Etoposide; CBDCA: Carboplatin; THP: Pirarubicin hydrochloride; CBA: Cyclophosphamide; EDTA: Ethylenediamine-tetraacetic acid; HE: Hematoxylin-eosin; PBS: Phosphate buffered saline; ISCN: International System for Human Cytogenetic Nomenclature; AFP: α-feto-protein; HCG: Human chorionic gonadotropin; SCC: Squamous cell carcinoma; TPA: Tissue polypeptide antigen; UIP: Universal Immuno-enzyme Polymer; DAB: Diaminobenzidine; ACD: Actinomycin D; ADM: Doxorubicin; 5-FU: 5-fluorouracil; MMC: Mitomycin C; BLM: Bleomycin; VLB: Vinblastine; VCR: Vincristine; PTX: Paclitaxel; CPT-11: Irinotecan SN-38; MTX: Methotrexate; MTT: 3-(4,5-dimethyl-2-thiazolyl)-2,5-diphenyl-2H tetrazolium bromide; DMSO: Dimethyl sulfoxide; EC50: Effective concentration for 50% kill.

## Competing interest

The authors declare that they have no competing interests.

## Authors’ contributions

TY and TK carried out surgery of the patient and drafted the manuscript. KS and MK examined microscopically surgical specimen and performed the immunohistochemical staining of the tumor. HM and YS contributed to the final data analysis and drafted the manuscript. All authors read and approved the final manuscript.
